# Zika Virus Infection, Basic and Clinical Aspects: A Review Article

**Published:** 2019-01

**Authors:** Farshid NOORBAKHSH, Kamal ABDOLMOHAMMADI, Yousef FATAHI, Hossein DALILI, Mehrnaz RASOOLINEJAD, Farshid REZAEI, Mostafa SALEHI-VAZIRI, Nazanin Zahra SHAFIEI-JANDAGHI, Ehsan Shamsi GOOSHKI, Morteza ZAIM, Mohammad Hossein NICKNAM

**Affiliations:** 1. Department of Immunology, School of Medicine, Tehran University of Medical Sciences, Tehran, Iran; 2. Department of Immunology, Faculty of Medicine, Kurdistan University of Medical Sciences, Sanandaj, Iran; 3. Department of Stem Cell Biology, Stem Cell Technology Research Center, Tehran, Iran; 4. Department of Pharmaceutical Nanotechnology, School of Pharmacy, Tehran University of Medical Sciences, Tehran, Iran; 5. Nanotechnology Research Center, School of Pharmacy, Tehran University of Medical Sciences, Tehran, Iran; 6. Department of Pediatrics, Breastfeeding Research Center, Tehran University of Medical Sciences, Tehran, Iran; 7. Department of Infectious Diseases, School of Medicine, Tehran University of Medical Sciences, Tehran, Iran; 8. Center for Control of Communicable Diseases, Ministry of Health and Medical Education, Tehran, Iran; 9. Department of Arboviruses and Viral Hemorrhagic Fevers (National Reference Laboratory), Pasteur Institute of Iran, Tehran, Iran; 10. Department of Virology, School of Public Health, Tehran University of Medical Sciences, Tehran, Iran; 11. Department of Medical Ethics, School of Medicine, Tehran University of Medical Sciences, Tehran, Iran; 12. Medical Ethics and History of Medicine Research Center, Tehran University of Medical Sciences, Tehran, Iran; 13. Department of Medical Entomology and Vector Control, School of Public Health, Tehran University of Medical Sciences, Tehran, Iran; 14. Molecular Immunology Research Center, Tehran University of Medical Sciences, Tehran, Iran

**Keywords:** Zika virus, Flaviviridae, Neurological infections, Fetal development, Microcephaly

## Abstract

**Background:**

Zika virus infection has recently attracted the attention of medical community. While clinical manifestations of the infection in adult cases are not severe and disease is not associated with high mortality rates, Zika virus infection can have an impact on fetal development and lead to severe neurodevelopmental abnormalities.

**Methods:**

To gain insight into different aspects of Zika virus infection, a comprehensive literature review was performed. With regard to epidemiology and geographical distribution of Zika virus infection, relevant information was extracted from CDC and WHO websites.

**Results:**

In this review, we discuss different basic and clinical aspects of Zika virus infection including virology, epidemiology and pathogenesis of disease. Laboratory methods required for the diagnosis of disease together with ethical issues associated with Zika virus infection will also be discussed in detail.

**Conclusion:**

Herein, we have tried to provide a multi-faceted view of Zika virus infection, with greater emphasis on disease status in Eastern Mediterranean Region.

## Introduction

Flaviviruses are responsible for several important human diseases including Dengue, West Nile and Yellow fevers, Japanese encephalitis, Tick-borne encephalitis, and Zika fever. The major route of transmission for most Flaviviruses is through arthropod vectors and central nervous system injury and hemorrhagic fevers represent major clinical outcomes. In recent years, Zika virus infections have attracted the attention of international medical community, chiefly because of their role in causing microcephaly and other neurodevelopmental abnormalities which occur as a consequence of maternal infections (1).

Zika virus was first isolated in 1947 in the Zika forest in Uganda from a rhesus macaque (2). Serological studies performed on human serum samples in Uganda later showed the presence of neutralizing antibodies against the virus, providing the first evidence that the virus can infect humans (3). Later studies revealed the presence of infection in other regions of Africa as well as Asian countries (4). Until 2007 Zika virus infections were mostly considered an infection of limited geographical distribution; however, later outbreaks of infection in Pacific islands and South America, associated with reports that the virus might cause nervous system abnormalities in newborns attracted the attention of a wide range of people in the scientific community. The situation reached its peak in 2015 when a striking increase occurred in reports of Zika virus infections in Brazil (5). The outbreak then spread to other countries in South, Central, and North America. The infections were associated with a significant increase in the number of microcephaly and Guillain–Barré syndrome cases in the infected regions, and this led to the declaration of a Public Health Emergency of International Concern (PHEIC) by the WHO in early 2016 (6–8). Since then, a strong international effort has been started to investigate epidemiological, molecular, pathogenic and clinical aspects of Zika virus infections.

**Fig. 1:**
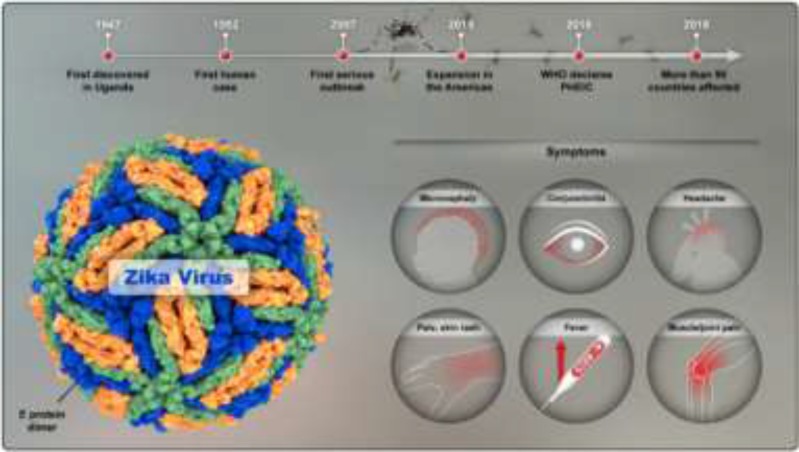
Zika virus infection at a glance (Original)

In this review, we have discussed the current knowledge about various aspects of Zika virus infection and recent developments including antiviral treatments for this infection. The first four sections of this review cover virology, epidemiology, pathogenesis, and laboratory diagnosis of Zika virus infection. While Zika virus infection is a global concern, in the epidemiology section we have put some more emphasis on findings from Eastern Mediterranean Region (EMRO) countries. Considering the ethical considerations related to feto-maternal and neurodevelopmental dimensions of the Zika virus infection, the final section of this review provides a detailed account of ethical aspects of Zika virus infection (Fig. 1).

### Virology of the Zika virus

Zika virus is a new emerging mosquito-borne virus belonging to the *Flaviviridae* family of viruses (9). This family is comprised of 4 genera: Flavivirus, Hepacivirus, Pegivirus, and Pestivirus (10). Zika virus belongs to the Flavivirus genus, which antigenically and phylogenetically is related to the Spondweni virus (9, 11). Many important human pathogens are included in this genus, for instance, Dengue, West Nile, Yellow fever, tick-borne encephalitis, Japanese encephalitis, Murray Valley encephalitis and St. Louis encephalitis viruses. These viruses are associated with a range of infections from asymptomatic or self-limiting febrile infections to some fatal diseases such as hemorrhage, shock, meningitis, and encephalitis (12).

In *Flaviviridae* family, all members have enveloped viruses with a single-stranded RNA genome of positive polarity (10) which contained one open reading frame (ORF) with two flanking noncoding regions (at 5′ and 3′ end) (13). The genomes are 5′ capped without a 3′ poly (A) tail.

A polyprotein is coded by the ORF then processed into three structural proteins (the envelope (E), the capsid (C) and the precursor of the membrane (prM)) and seven nonstructural proteins (NS1, NS2A, NS2B, NS3, NS4A, NS4B, and NS5) (1, 12). The ssRNA is held within an icosahedral capsid shaped from 12-kDa protein blocks; the nucleocapsid is surrounded by a host-derived membrane contained two viral glycoproteins (14).

Similar replication strategies are employed by the members of *Flaviviridae* family despite significant differences in tissue tropism, transmission, and pathogenesis (10).

### Recent developments in Zika antiviral treatments

At the present time, no effective antiviral treatments are available for Zika virus infection (15). However, considering rapid geographic expansion of Zika virus, its severe neurological complications and devastating effects on the fetus, investigations are underway to find safe and potent drugs and therapies. These approaches range from repurposing earlier approved drugs to the screening and testing of in silico designed drugs (16). The antiviral candidates comprise agents targeting cellular components in addition to viral ones (15). All of the steps of Zika virus life cycle in the host cell from entry to release can be targeted by antiviral agents.

In order to block the viral entry into the cell, various strategies have been used. On one hand, blocking the entry by receptor binding agents such as nanchangmycin (previously used as antibacterial and insecticidal agent), ZINC33683341 and ZINC49605556 (both in silico designed) has been proposed. The other approaches include the inhibition of endosomal fusion using compounds that reduce the acidity of endolysosomal vesicles like chloroquine (an anti-malaria drug) and disrupting the electrostatic interaction between cell and virus membrane using squalamine (a cationic chemical) (16).

RdRP activity of NS5 is an outstanding target for antiviral drugs. Therefore, several nucleoside analogues are investigated for their ability to inhibit Zika virus replication. Despite demonstrated efficacy of these drugs in cell cultures and animal models, some of them were unsuccessful in the clinical trials. Sofosbuvir (a nucleotide analog inhibitor) is a RdRP inhibitor approved by FDA for chronic HCV treatment. It also inhibits Zika virus replication (15–17). Zika virus N2B-NS3 protease and NS3 helicase, which play essential roles in virus replication, constitute potential targets for antiviral drugs (15). As the Zika virus helicase is similar to those of the other members of *Flaviviridae*, antiviral agents targeted their helicase could also be used for the Zika virus (18). Likewise, berberine previously used against dengue virus showed a high binding affinity to the Zika virus NS3 protease (19) as well.

Despite the many studies done on Zika antiviral treatments, so far no FDA approved category A drug has been found safe to use in mothers and fetuses (16).

### Epidemiology of Zika virus infection

Before the first large outbreak of Zika virus infection on Yap Island, Federated States of Micronesia (20), only sporadic cases and serological evidence of Zika virus were reported in western and central Africa and south-east Asia (21, 22), but in 2007 Zika Virus emerged as an important human pathogen.

An increased incidence of cases of the Guillain– Barré syndrome (GBS) was reported after a larger epidemic of Zika virus infection in French Polynesia in 2013 (23). In 2014, autochthonous transmission of Zika virus infection occurred in Easter Island (Chile) from Feb until Jul (24).

In early 2015, several cases presenting a “dengue-like syndrome” investigated in Brazil (a non-Dengue virus and non-Chikungunya virus infection) and Zika virus was detected by reverse transcription polymerase chain reaction (RT-PCR) assay and confirmed by DNA sequencing (25, 26). The Brazil Zika virus strain shares a common ancestor with the Zika virus strain that circulated in French Polynesia (27).

On Jul 2015, in the State of Bahía, Brazil, an increase in the number of Guillain-Barré cases in which half of them had reported symptoms consistent with Zika virus infection (28).

In Jan 2016, an unusually significant increase of GBS was reported in El Salvador. An Emergency Committee was convened by the Director-General of WHO, under the International Health Regulations (2005) on 1 Feb 2016, and finally announced “the recent cluster of microcephaly and other neurologic disorders reported in Brazil to be a PHEIC (28).

### Situation and risk analysis in Eastern Mediterranean Region, regional strategic plan

**Fig. 2:**
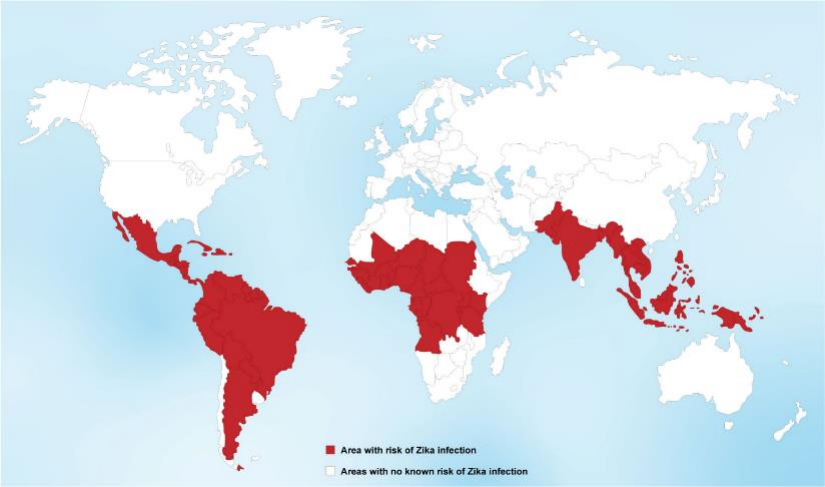
Global map of Zika virus infection

Although no countries of the Eastern Mediterranean Region of WHO have reported importation of Zika virus disease or autochthonous transmission, however, of the 22 countries in the Region, the following 8 have reported dengue outbreaks in recent years and/or have the presence of competent *Aedes* mosquitoes: Djibouti, Egypt, Oman, Pakistan, Saudi Arabia, Somalia, Sudan and Yemen. Some of these countries are particularly vulnerable to emerging of Zika virus, because of the fragility of health systems, weakness of disease surveillance systems, the inadequacy of response capacities, and a suboptimal level of public health preparedness (29–31). Pakistan is the most-at-risk country in the Region, primarily due to a large number of travelers to and from the Americas, dense urban populations, a documented large epidemic of dengue, and the most favorable climatic conditions for the reproduction of *Aedes* mosquitoes (29). In Iran, national committee of *Aedes*-borne diseases recommended enhancement of surveillance after 2016 and updated national guidelines of surveillance and clinical management of suspected cases, especially for travelers returning from at-risk countries. As of Sep 2018, all of the samples from suspected cases of Zika virus sent to the National Laboratory of *Aedes-*borne diseases were shown to be negative. Prevention of *Aedes*-borne diseases needs comprehensive national strategic action plan with “one health” approach and close collaboration between community, academic, and public health authorities. Global map of Zika virus infection demonstrated in (Fig. 2) and areas potentially at risk of Zika in (Table 1).

**Table 1:** Areas and countries potentially at risk of Zika

***Africa***	Angola, Benin, Burkina-Faso, Burundi, Cameroon, Cape Verde, Central African Republic, Chad, Congo (Congo-Brazzaville), Côte d’Ivoire, Democratic Republic of the Congo (Congo-Kinshasa), Equatorial Guinea, Gabon, Gambia, Ghana, Guinea, Guinea-Bissau, Kenya, Liberia, Mali, Niger, Nigeria, Rwanda, Senegal, Sierra Leone, South Sudan, Sudan, Tanzania, Togo, Uganda
***Asia***	Bangladesh, Burma (Myanmar), Cambodia, India, Indonesia, Laos, Malaysia, Maldives, Pakistan, Philippines, Singapore, Thailand, Timor-Leste (East Timor), Vietnam
***The Caribbean***	Anguilla; Antigua and Barbuda; Aruba; Barbados; Bonaire; British Virgin Islands; Cuba; Curaçao; Dominica; Dominican Republic; Grenada; Haiti; Jamaica; Montserrat; the Commonwealth of Puerto Rico, a US territory; Saba; Saint Kitts and Nevis; Saint Lucia; Saint Martin; Saint Vincent and the Grenadines; Sint Eustatius; Sint Maarten; Trinidad and Tobago; Turks and Caicos Islands; US Virgin Islands
***Central America***	Belize, Costa Rica, El Salvador, Guatemala, Honduras, Nicaragua, Panama
***North America***	Mexico
***The Pacific Islands***	Fiji, Papua New Guinea, Samoa, Solomon Islands, Tonga
***South America***	Argentina, Bolivia, Brazil, Colombia, Ecuador, French Guiana, Guyana, Paraguay, Peru, Suriname, Venezuela

Content source: Centers for Disease Control and Prevention, September 2018 updated. ( https://wwwnc.cdc.gov/travel/page/world-map-areas-with-zika )

Content source: Centers for Disease Control and Prevention, Sep 2018 updated. ( https://wwwnc.cdc.gov/travel/page/world-map-areas-with-zika )

### Pathogenesis of Zika virus infection

**Fig. 3:**
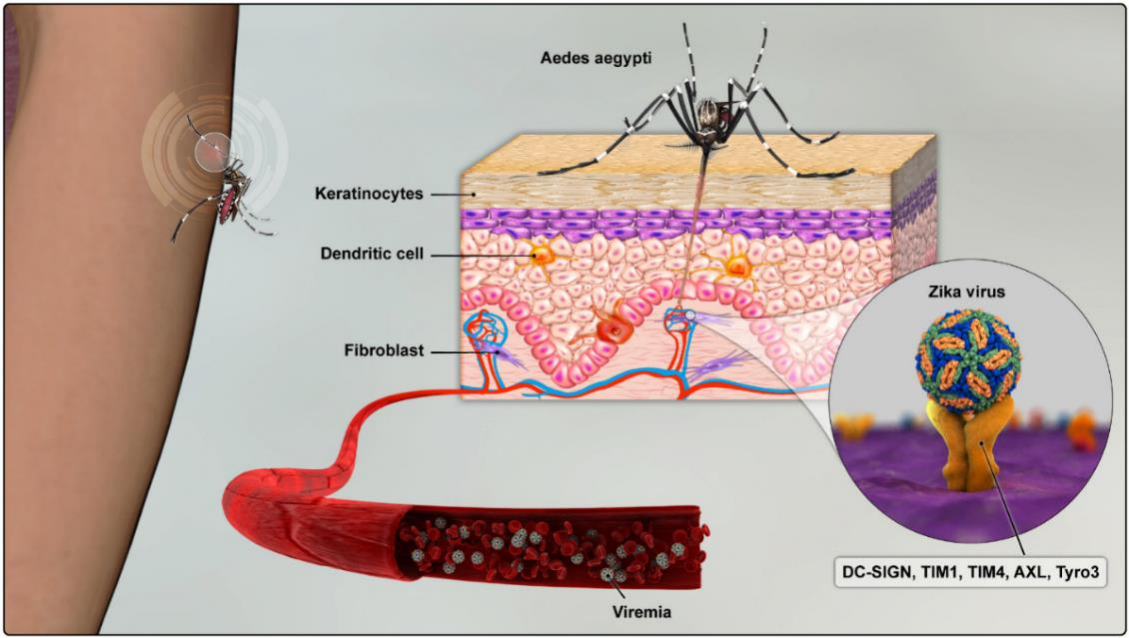
Immunopathogenic pathways and virus-host cell interaction in Zika virus infection. Infected vectors (*Aedes aegypti* and *Aedes albopictus*) introduce Zika viruses into the host during their blood meal. A variety of cells (keratinocytes, fibroblasts, and immature dendritic cells) can be infected through receptor-mediated endocytosis using flaviviruses E glycoprotein. Zika virus entry into these cells is mediated by several receptors including DC-SIGN (CD209), TIM-1, 4 (T-cell immunoglobulin and mucin domain-1, 4), AXL and Tyro3 (cell surface receptor tyrosine kinases, part of the TAM family) (Original)

Studies on the pathogenesis of Zika virus have shown similarities with the pathogenesis of other Flavivirus infections. Following transmission by the mosquito bite, Zika virus can infect several different cell types including skin keratinocytes, dermal fibroblasts and dendritic cells (DCs) (9). In vitro studies on fibroblasts exposed to Zika virus have shown high infection rates in these cells 24 to 48 h after infection. Flow cytometric analyses of DCs exposed to the virus have also shown that up to 60% of DCs express viral antigens around 24 h after infection (9). Different cell surface receptors have been proposed to mediate Zika infection of permissive cells; including DC-SIGN, AXL and Tyro3 molecules (9) (Fig. 3).

Following cell entry, Zika virus induces strong interferon responses in infected cells. Studies on Zika virus-infected human primary fibroblasts have shown strong upregulation of interferon-beta transcripts 24 to 48 h after infection (9). A milder response has been reported for interferon-alpha. Upregulation of intracellular “pattern recognition receptors” (PRRs) involved in sensing non-self-nucleic acids seems to be a crucial part of the initial immune response to the virus (9). Studies on Zika virus-infected fibroblasts have revealed strong induction of RIG-I and MDA-5 transcripts. Both of these molecules are capable of initiating a signaling process following the detection of intracytoplasmic viral RNA molecules. Innate immune responses are followed by adaptive immune events including the activation of T cells; Zika virus-infected DCs migrate to regional lymph nodes where they stimulate T cell proliferation, differentiation and cytokine production (32).

Productive infection of dermal fibroblasts and dendritic cells together with insufficient control of infection by innate and adaptive immune mechanisms usually leads to Zika virus viremia, which underlies the non-specific clinical symptoms which might last for a few days. In a pregnant woman, maternal viremia might lead to fetal viremia. It is not well known how the virus is transmitted to developing nervous system following the establishment of fetal viremia. While in circulation, Zika virus can infect fetal monocytes and these monocytes can carry the infection to the developing nervous system. Studies on fetal brain tissue derived from Zika virus-infected mothers who have undergone abortion have shown viral particles in neural cells, indicating that the virus can proliferate inside neural cells in a developing brain (5). While inside the CNS, Zika virus can exert direct neurovirulence, similar to other Flaviviruses. There is also evidence that Zika virus might cause indirect neurovirulence through the activation of immune mechanisms including microglial activation and macrophage infiltration (5). Indeed, in adults Zika virus might be an etiological factor for “Acute Disseminated Encephalomyelitis” (ADEM) (33), an immune-mediated disease of the CNS, which occurs subsequent to varieties of viral and nonviral infections (34). Whether an ADEM-like process might affect developing brain remains an open question.

### Laboratory diagnosis of Zika virus infection

Depending on the purpose of the investigation, laboratory diagnosis of Zika virus can be conducted by virus isolation, antigen detection, viral RNA detection with molecular assays and anti-Zika virus antibodies detection with serological assays (35).

### Virus Isolation

The first isolation of Zika virus was performed by intracerebral mouse inoculation considered as the reference assay for arboviruses isolation (25, 36). Among human clinical specimens, Zika virus can be cultured from blood (37), urine (38), saliva (39) and semen (40).

### Antigen Detection

Antigen detection is a valuable assay to approved Zika virus infection in autopsy tissues. Using immunohistochemistry (IHC) technique, Zika virus antigen has been detected in brain and placental tissues from congenitally infected newborns with microcephaly and miscarriages (41, 42). Recently, new assays for detection of Zika virus antigen including NS3 protein identification by flow cytometry in whole blood (43), aptamer-based ELISA assay for targeting of Zika virus NS1 protein (44), NS1 protein based competitive ELISA (45), NS1 protein–based rapid tests (46) have been developed.

### Molecular Assays

Zika virus RNA is detectable in different types of body fluids such as blood (serum or plasma) (47–50), urine (49, 51), saliva (52) semen (40), breast milk (49), conjunctival fluid (53) and amniotic fluid (54); and brain and placental tissues of congenitally infected fetuses (41, 42).

Reverse transcriptase PCR (RT-PCR) is highly sensitive and specific and known as the “gold standard” for ZIKV diagnosis (55). Regarding Zika virus-specific RT-PCR, several conventional and real-time assays targeting prM, E, NS1, NS3, NS4, and NS5 genes have been developed (50, 56–59). However, to the best of our knowledge, the Food and Drug Administration (FDA) has approved only one commercial assay e.g. Cobas Zika test (Roche) which is a qualitative nucleic acid test for screening Zika virus RNA in blood donors. Additionally, FDA has authorized the use of several molecular assays under an Emergency Use Authorization (EUA) (60) all of them are based on RT-PCR (such as Triplex Real-Time RT-PCR, a multiplex assay for detection of Zika virus, Dengue virus, and Chikungunya virus (CDC) (61), Zika virus RNA qualitative real-time RT-PCR (Quest Diagnostics Infectious Disease, Inc.) and RealStar Zika virus RT-PCR kit (Altona Diagnostics, GmbH)) expect Aptima Zika virus assay based on Transcription-Mediated Amplification (TMA) Technology (60).

Molecular diagnosis of Zika virus infection in human usually performs on plasma or serum specimens within the first week after onset of clinical symptoms (60). Although, there are several lines of evidence for advantage of urine for Zika virus RNA detection because of the long duration of viral shedding in this easily collectable specimen (55 , 12, 62), interesting findings indicating the shorter persistence of ZIKV RNA in urine versus serum have been observed (60, 63, 64).

### Serological Assays

Despite the fact that the molecular diagnosis possesses high sensitivity and specificity, short period of viremia can negatively affect the Zika virus RNA detection (65). Therefore, detection of anti-Zika virus antibodies by serological assays could be an advantageous option for a wider diagnostic window.

Detection of anti-Zika virus antibodies can be performed by different serological tests including complement fixation, haemagglutination inhibition, immunofluorescence (IF) assay, ELISA and neutralization tests (13, 35). Typically, anti-Zika virus IgM antibody develops within the first week after the onset of symptoms and is detectable from day five to 12 wk of illness. Anti Zika virus IgG antibody rises few days after IgM and is traceable for months to years.

So far there is no FDA approved a serological test for Zika virus. However, FDA has authorized the use of five serological assays with emergency use authorization for detection of anti-Zika virus IgM antibody including IgM antibody capture ELISA (Zika MAC-ELISA) (CDC) (61), ZIKV Detect IgM capture ELISA (InBios International, Inc.), Liaison XL Zika capture IgM assay (DiaSorin Incorporated), ADVIA Centaur Zika test (Siemens Healthcare Diagnostics Inc.) and DPP Zika IgM system (Chembio Diagnostic Systems, Inc.) (60).

Because of cross-reactivity with other Flaviviruses such as dengue and yellow fever viruses, and the possibility of nonspecific reactivity, results of IgM detection assays should be interpreted with caution and positive or equivocal results must be confirmed by plaque-reduction neutralization testing (PRNT) (66).

### Laboratory Biosafety

Zika virus has been classified as a risk group 2 human pathogen. Therefore, diagnostic laboratory tests should be performed in a Biosafety Level 2 (BSL-2) facility (13). The virus can be inactivated by Ultraviolet (UV) radiation, temperatures above 58 °C, solutions of pH under 6.2 above < 7.8, ether, and 5% potassium permanganate (67–69).

### Ethical aspects and challenges of Zika virus infection

Addressing ethical questions and moral conflicts of Zika virus infection may be essential to develop a comprehensive approach to control this public health crisis (70). Therefore, the health authorities commitment to allocating a fair portion of health resources for responding to this situation is morally required (71). The government should priorities paying the noticeable amount of money for preventive measures in order to control the Zika virus spread (72). This priority setting is usually the most difficult step while encountering such situations, especially is the absence of enough scientific evidence to measure the real burden of disease and also the presence of social and media pressure, amplified by 2016 Olympic games in Brazil (73).

National and international health authorities should observe the principle of veracity, by clear and transparent informing system for raising the public awareness regarding the real situation (74). At the international level, the “Cosmopolitan Solidarity” for encountering such universal pattern of disease spread seems to be inevitable (75). Like Ebola outbreak, responsiveness to actions required by responsible international bodies especially WHO is another responsibility of states to form a global health governance network (76). In mosquito control, governments should try to keep the moral obligation of observing biosafety and protecting the ecosystems in the mind while trying to restrict the insect spread and reproduction (77). During policy-making for disease control, deliberative ethical evaluation is required in order to protect of population basic civil and human rights such as people’s free mobilization or reproductive rights in case that border or birth control are among the solutions (78, 79). Similarly, confidentiality of patients’ health information and protecting people against stigmatization must be observed when policies for reporting the probable cases to health authorities are discussed (80).

The fear raised from high probability of congenital problems, mainly microcephaly, of neonates delivered by mothers with Zika virus infection in their first trimester of pregnancy (81), puts abortion at the center of moral concerns (82). The position of various schools of ethics regarding abortion has a range from strict prohibition to complete moral justification based on the moral status they consider for the fetus in each development stage. Accordingly, abortion laws are still different in various countries according to the social, political, religious and cultural context (83, 84). In Iran despite of general prohibition of abortion by Criminal Law, the “Therapeutic Abortion Act” of 2005 allowed performing abortion in presence of a “definite diagnosis of retardation or malformation of the fetus that is unbearable for the mother…” only before 4 months (19th wk) after conception and any attempt for abortion after this gestational age is illegal and even criminal (85).

## Conclusion

Together with Severe Acute Respiratory Syndrome (SARS) and Middle East Respiratory Syndrome (MERS), Zika virus infection qualifies as a “newly emerging” infectious disease, with the potential to cause serious public health issues. Unlike the other “newly emerging” infections which can lead to severe morbidity and mortality in infected adults or pediatric hosts, Zika infection does not pose a significant threat to infected adults and its risk is more due to the potential to cause fetal abnormalities, provided that the infection occurs during pregnancy. Indeed, among the four recent PHEIC declarations by WHO (i.e. 2009 Swine flu declaration, 2014 Polio and Ebola declarations and 2016 Zika virus declaration), Zika is unique in the sense that it is a member of the TORCH group of infections; i.e. the group of pathogens with the ability to lead to congenital infections/anomalies. This might influence both disease prevention and management strategies as well as raising ethical and sociological issues. Thus, increased awareness of the medical community together with improvements in vector control and disease surveillance systems are of utmost importance for controlling any potential Zika virus-related threats in different countries.

## Ethical considerations

Ethical issues (Including plagiarism, informed consent, misconduct, data fabrication and/or falsification, double publication and/or submission, redundancy, etc.) have been completely observed by the authors.

## References

[1] Kuno G Chang G-JJ Tsuchiya KR (1998). Phylogeny of the genus Flavivirus. J Virol, 72( 1): 73– 83. 9420202 10.1128/jvi.72.1.73-83.1998 PMC109351

[2] Simpson D (1964). Zika virus infection in man. Trans R Soc Trop Med Hyg, 58: 335– 8. 14175744

[3] Dick GW (1952). Zika Virus (II). Pathogenicity and physical Properties. Trans R Soc Trop Med Hyg, 46 ( 5): 521– 34. 12995441 10.1016/0035-9203(52)90043-6

[4] Wikan N Smith DR (2016). Zika virus: history of a newly emerging arbovirus. Lancet Infect Dis, 16( 7): e119– e126. 27282424 10.1016/S1473-3099(16)30010-X

[5] Mlakar J Korva M Tul N (2016). Zika virus associated with microcephaly. N Engl J Med, 374( 10): 951– 8. 26862926 10.1056/NEJMoa1600651

[6] Sikka V Chattu VK Popli RK (2016). The emergence of Zika virus as a global health security threat: a review and a consensus statement of the INDUSEM Joint Working Group (JWG). J Glob Infect Dis, 8( 1): 3– 15. 27013839 10.4103/0974-777X.176140 PMC4785754

[7] WHO (2016). WHO Director-General summarizes the outcome of the Emergency Committee regarding clusters of microcephaly and Guillain-Barré syndrome.

[8] Heymann DL Hodgson A Sall AA (2016). Zika virus and microcephaly: why is this situation a PHEIC? Lancet, 387( 10020): 719– 21. 26876373 10.1016/S0140-6736(16)00320-2 PMC7134564

[9] Hamel R Dejarnac O Wichit S (2015). Biology of Zika virus infection in human skin cells. J Virol, 89( 17): 8880– 96. 26085147 10.1128/JVI.00354-15 PMC4524089

[10] Paul D Bartenschlager R (2015). Flaviviridae replication organelles: oh, what a tangled web we weave. Annu Rev Virol, 2( 1): 289– 310. 26958917 10.1146/annurev-virology-100114-055007

[11] Marano G Pupella S Vaglio S (2016). Zika virus and the never-ending story of emerging pathogens and transfusion medicine. Blood Transfus, 14( 2): 95– 100. 26674815 10.2450/2015.0066-15 PMC4786129

[12] Muller DA Young PR (2013). The flavivirus NS1 protein: molecular and structural biology, immunology, role in pathogenesis and application as a diagnostic biomarker. Antiviral Res, 98( 2): 192– 208. 23523765 10.1016/j.antiviral.2013.03.008

[13] Musso D Gubler DJ (2016). Zika virus. Clin Microbiol Rev, 29( 3): 487– 524. 27029595 10.1128/CMR.00072-15 PMC4861986

[14] Pierson TC Diamond MS (2012). Degrees of maturity: the complex structure and biology of flaviviruses. Curr Opin Virol, 2( 2): 168– 75. 22445964 10.1016/j.coviro.2012.02.011 PMC3715965

[15] Saiz J-C Martín-Acebes MA (2017). The race to find antivirals for Zika virus. Antimicrob Agents Chemother, 61( 6): e00411– 17. 28348160 10.1128/AAC.00411-17 PMC5444186

[16] Munjal A Khandia R Dhama K (2017). Advances in developing therapies to combat Zika virus: current knowledge and future perspectives. Front Microbiol, 8: 1469. 28824594 10.3389/fmicb.2017.01469 PMC5541032

[17] Khodakhah F Mokhtari Azad T (2018). A review on Zika virus, a re-emerging arbovirus. Tehran Univ Med J, 75( 11): 779– 89.

[18] Tian H Ji X Yang X (2016). The crystal structure of Zika virus helicase: basis for antiviral drug design. Protein Cell, 7( 6): 450– 4. 27172988 10.1007/s13238-016-0275-4 PMC4887331

[19] Sahoo M Jena L Daf S (2016). Virtual screening for potential inhibitors of NS3 protein of Zika virus. Genomics Inform, 14( 3): 104– 111. 27729840 10.5808/GI.2016.14.3.104 PMC5056895

[20] Duffy MR Chen T-H Hancock WT (2009). Zika virus outbreak on Yap Island, federated states of Micronesia. N Engl J Med, 360( 24): 2536– 43. 19516034 10.1056/NEJMoa0805715

[21] Fagbami AH (1979). Zika virus infections in Nigeria: virological and seroepidemiological investigations in Oyo State. J Hyg (Lond), 83( 2): 213– 9. 489960 10.1017/s0022172400025997 PMC2129900

[22] Moore DL Causey OR Carey DE (1975). Arthropod-borne viral infections of man in Nigeria, 1964–1970. Ann Trop Med Parasitol, 69( 1): 49– 64. 1124969 10.1080/00034983.1975.11686983

[23] Oehler E Watrin L Larre P (2014). Zika virus infection complicated by Guillain-Barre syndrome--case report, French Polynesia, December 2013. Euro Surveill, 19( 9): 20720. 24626205 10.2807/1560-7917.es2014.19.9.20720

[24] PAHO (2016). Timeline of Emergence of Zika virus in the Americas. Pan American Health Organization. https://www.paho.org/hq/index.php?option=com_content&view=article&id=11959:timeline-of-emergence-of-zika-virus-in-the-americas&Itemid=41711&lang=en

[25] Campos GS Bandeira AC Sardi SI (2015). Zika virus outbreak, Bahia, Brazil. Emerg Infect Dis, 21( 10): 1885– 6. 26401719 10.3201/eid2110.150847 PMC4593454

[26] Zanluca C Melo VC Mosimann AL (2015). First report of autochthonous transmission of Zika virus in Brazil. Mem Inst Oswaldo Cruz, 110( 4): 569– 72. 26061233 10.1590/0074-02760150192 PMC4501423

[27] Musso D (2015). Zika virus transmission from French Polynesia to Brazil. Emerg Infect Dis, 21( 10): 1887. 10.3201/eid2110.151125 PMC4593458 26403318

[28] WHO (2016). Zika situation report 5 February 2016. https://www.who.int/emergencies/zika-virus/situation-report/5-february-2016/en/

[29] WHO (2017). Zika preparedness plan for the Eastern Mediterranean Region. http://www.emro.who.int/health-topics/zika/zika-preparedness-plan-for-the-eastern-mediterranean-region.html

[30] Gavlak D (2015). Health system in Yemen close to collapse: Yemen is facing a growing humanitarian catastrophe as health workers there risk their lives to help civilians caught up in the deadly conflict. Bull World Health Organ, 93( 10): 670– 671. 26600607 10.2471/BLT.15.021015 PMC4645439

[31] Shrivastava S Shrivastava P Ramasamy J (2017). World Health Organization raises concern over the urgent need to respond to the multiple outbreaks of infectious diseases reported in South Sudan amidst the ongoing conflict. ATMPH, 10( 6): 1407– 8.

[32] Tappe D Pérez-Girón JV Zammarchi L (2016). Cytokine kinetics of Zika virus-infected patients from acute to reconvalescent phase. Med Microbiol Immunol, 205( 3): 269– 73. 26702627 10.1007/s00430-015-0445-7 PMC4867002

[33] Niemeyer B Niemeyer R Borges R (2017). Acute Disseminated Encephalomyelitis Following Zika Virus Infection. Eur Neurol, 77( 1–2): 45– 46. 27894121 10.1159/000453396

[34] Noorbakhsh F Johnson RT Emery D (2008). Acute disseminated encephalomyelitis: clinical and pathogenesis features. Neurol Clin, 26( 3): 759– 80. 18657725 10.1016/j.ncl.2008.03.009 PMC7132764

[35] Zanluca C dos Santos CND (2016). Zika virus– an overview. Microbes Infect, 18( 5): 295– 301. 26993028 10.1016/j.micinf.2016.03.003

[36] Dick GW Kitchen SF Haddow AJ (1952). Zika virus (I). Isolations and serological specificity. Trans R Soc Trop Med Hyg, 46( 5): 509– 20. 12995440 10.1016/0035-9203(52)90042-4

[37] Hancock WT Marfel M Bel M (2014). Zika virus, French polynesia, South pacific, 2013. Emerg Infect Dis, 20( 6): 1085– 6. 25341051 10.3201/eid2011.141253 PMC4214323

[38] Kutsuna S Kato Y Takasaki T (2014). Two cases of Zika fever imported from French Polynesia to Japan, December 2013 to January 2014. Euro Surveill, 19( 4): 20683. 24507466 10.2807/1560-7917.es2014.19.4.20683

[39] Bonaldo MC Ribeiro IP Lima NS (2016). Isolation of Infective Zika Virus from Urine and Saliva of Patients in Brazil. PLoS Negl Trop Dis, 10( 6): e0004816. 27341420 10.1371/journal.pntd.0004816 PMC4920388

[40] Musso D Roche C Robin E (2015). Potential sexual transmission of Zika virus. Emerg Infect Dis, 21( 2): 359– 61. 25625872 10.3201/eid2102.141363 PMC4313657

[41] Martines RB Bhatnagar J Keating MK (2016). Notes from the field: evidence of Zika virus infection in brain and placental tissues from two congenitally infected newborns and two fetal losses—Brazil, 2015. MMWR Morb Mortal Wkly Rep, 65( 6): 159– 60. 26890059 10.15585/mmwr.mm6506e1

[42] Martines RB Bhatnagar J de Oliveira Ramos AM (2016). Pathology of congenital Zika syndrome in Brazil: a case series. Lancet, 388( 10047): 898– 904. 27372395 10.1016/S0140-6736(16)30883-2

[43] Lum FM Lin C Susova OY (2017). A Sensitive Method for Detecting Zika Virus Antigen in Patients’ Whole-Blood Specimens as an Alternative Diagnostic Approach. J Infect Dis, 216( 2): 182– 190. 28586426 10.1093/infdis/jix276 PMC5853302

[44] Lee KH Zeng H (2017). Aptamer-Based ELISA Assay for Highly Specific and Sensitive Detection of Zika NS1 Protein. Anal Chem, 89( 23): 12743– 12748. 29120623 10.1021/acs.analchem.7b02862

[45] Balmaseda A Stettler K Medialdea-Carrera R (2017). Antibody-based assay discriminates Zika virus infection from other flaviviruses. Proc Natl Acad Sci U S A, 114( 31): 8384– 8389. 28716913 10.1073/pnas.1704984114 PMC5547631

[46] Bosch I de Puig H Hiley M (2017). Rapid antigen tests for dengue virus serotypes and Zika virus in patient serum. Sci Transl Med, 9( 409): 1589. 10.1126/scitranslmed.aan1589 PMC6612058 28954927

[47] Zammarchi L Stella G Mantella A (2015). Zika virus infections imported to Italy: clinical, immunological and virological findings, and public health implications. J Clin Virol, 63: 32– 5. 25600600 10.1016/j.jcv.2014.12.005

[48] Waehre T Maagard A Tappe D (2014). Zika virus infection after travel to Tahiti, December 2013. Emerg Infect Dis, 20( 8): 1412– 4. 25062427 10.3201/eid2008.140302 PMC4111184

[49] Besnard M Lastere S Teissier A (2014). Evidence of perinatal transmission of Zika virus, French Polynesia, December 2013 and February 2014. Euro Surveill, 19( 13): 20751. 24721538

[50] Lanciotti RS Kosoy OL Laven JJ (2008). Genetic and Serologic Properties of Zika Virus Associated with an Epidemic, Yap State, Micronesia, 2007. Emerg Infect Dis, 14( 8): 1232– 9. 18680646 10.3201/eid1408.080287 PMC2600394

[51] Gourinat AC O’Connor O Calvez E (2015). Detection of Zika virus in urine. Emerg Infect Dis, 21( 1): 84– 6. 25530324 10.3201/eid2101.140894 PMC4285245

[52] Musso D Roche C Nhan TX (2015). Detection of Zika virus in saliva. J Clin Virol, 68: 53– 5. 26071336 10.1016/j.jcv.2015.04.021

[53] Tan JJL Balne PK Leo YS (2017). Persistence of Zika virus in conjunctival fluid of convalescence patients. Sci Rep, 7: 11194. 28894118 10.1038/s41598-017-09479-5 PMC5594005

[54] Oliveira Melo AS Malinger G Ximenes R (2016). Zika virus intrauterine infection causes fetal brain abnormality and microcephaly: tip of the iceberg? Ultrasound Obstet Gynecol, 47( 1): 6– 7. 26731034 10.1002/uog.15831

[55] Mauk MG Song J Bau HH (2017). Point-of-Care Molecular Test for Zika Infection. Clin Lab Int, 41: 25– 27. 28819345 PMC5556939

[56] Tappe D Rissland J Gabriel M (2014). First case of laboratory-confirmed Zika virus infection imported into Europe, November 2013. Euro Surveill, 19( 4): 20685. 24507467 10.2807/1560-7917.es2014.19.4.20685

[57] Faye O Faye O Diallo D (2013). Quantitative real-time PCR detection of Zika virus and evaluation with field-caught mosquitoes. Virol J, 10: 311. 24148652 10.1186/1743-422X-10-311 PMC4016539

[58] Faye O Faye O Dupressoir A (2008). One-step RT-PCR for detection of Zika virus. J Clin Virol, 43( 1): 96– 101. 18674965 10.1016/j.jcv.2008.05.005

[59] Balm MN Lee CK Lee HK (2012). A diagnostic polymerase chain reaction assay for Zika virus. J Med Virol, 84( 9): 1501– 5. 22825831 10.1002/jmv.23241

[60] Theel ES Hata DJ (2018). Diagnostic Testing for Zika Virus: a Postoutbreak Update. J Clin Microbiol, 56( 4): e01972– 17. 29386264 10.1128/JCM.01972-17 PMC5869840

[61] CDC (2017). Diagnostic Tests for Zika Virus. Centers for Disease Control and Prevention. https://www.cdc.gov/zika/hc-providers/types-of-tests.html

[62] Bingham AM Cone M Mock V (2016). Comparison of Test Results for Zika Virus RNA in Urine, Serum, and Saliva Specimens from Persons with Travel-Associated Zika Virus Disease - Florida, 2016. MMWR Morb Mortal Wkly Rep, 65( 18): 475– 8. 27171533 10.15585/mmwr.mm6518e2

[63] Paz-Bailey G Rosenberg ES Doyle K (2017). Persistence of Zika Virus in Body Fluids - Final Report. N Engl J Med, 379( 13): 1234– 1243. 28195756 10.1056/NEJMoa1613108 PMC5831142

[64] Brasil P Pereira JP Jr Moreira ME (2016). Zika Virus Infection in Pregnant Women in Rio de Janeiro. N Engl J Med, 375( 24): 2321– 2334. 26943629 10.1056/NEJMoa1602412 PMC5323261

[65] Singh RK Dhama K Karthik K (2018). Advances in Diagnosis, Surveillance, and Monitoring of Zika Virus: An Update. Front Microbiol, 8: 2677. 29403448 10.3389/fmicb.2017.02677 PMC5780406

[66] Rabe IB Staples JE Villanueva J (2016). Interim guidance for interpretation of Zika virus antibody test results. MMWR Morb Mortal Wkly Rep, 65( 21): 543– 6. 27254248 10.15585/mmwr.mm6521e1

[67] Chernesky M Castriciano S Mahony J (1985). Examination of the Rotazyme II enzyme immunoassay for the diagnosis of rotavirus gastroenteritis. J Clin Microbiol, 22( 3): 462– 4. 2995441 10.1128/jcm.22.3.462-464.1985 PMC268437

[68] Blumel J Musso D Teitz S (2017). Inactivation and removal of Zika virus during manufacture of plasma-derived medicinal products. Transfusion, 57( 3pt2): 790– 796. 27731495 10.1111/trf.13873

[69] Muller JA Harms M Schubert A (2016). Inactivation and Environmental Stability of Zika Virus. Emerg Infect Dis, 22( 9): 1685– 7. 27367466 10.3201/eid2209.160664 PMC4994368

[70] Saenz C (2016). Zika virus: ethics preparedness for old and new challenges. Lancet Glob Health, 4( 10): e686. 27633426 10.1016/S2214-109X(16)30222-4

[71] Barnett DJ Taylor HA Hodge JG Jr (2009). Resource allocation on the frontlines of public health preparedness and response: report of a summit on legal and ethical issues. Public Health Rep, 124( 2): 295– 303. 19320372 10.1177/003335490912400218 PMC2646457

[72] Alfaro-Murillo JA Parpia AS Fitzpatrick MC (2016). A cost-effectiveness tool for informing policies on Zika virus control. PLoS Negl Trop Dis, 10( 5): e0004743. 27205899 10.1371/journal.pntd.0004743 PMC4874682

[73] Baker DE (2016). Zika Virus and the Media. Hosp Pharm, 51( 4): 275– 6. 27303071 10.1310/hpj5104-275 PMC4896326

[74] Guttman N Salmon CT (2004). Guilt, fear, stigma and knowledge gaps: ethical issues in public health communication interventions. Bioethics, 18( 6): 531– 52. 15580723 10.1111/j.1467-8519.2004.00415.x

[75] Lencucha R (2013). Cosmopolitanism and foreign policy for health: ethics for and beyond the state. BMC Int Health Hum Rights, 13: 29. 23829176 10.1186/1472-698X-13-29 PMC3717113

[76] Kickbusch I Reddy KS (2015). Global health governance–the next political revolution. Public Health, 129( 7): 838– 42. 26040216 10.1016/j.puhe.2015.04.014

[77] Macer D (2005). Ethical, legal and social issues of genetically modifying insect vectors for public health. Insect Biochem Mol Biol, 35( 7): 649– 60. 15894183 10.1016/j.ibmb.2005.02.010

[78] Bueno MA Grunspun H (2016). Bioethical considerations at times of Zika virus. Einstein (Sao Paulo), 14( 2): 13– 8. 27462882 10.1590/S1679-45082016ED3725 PMC4943367

[79] Royo-Bordonada MÁ López FJG (2016). Ethical considerations surrounding the response to Ebola: the Spanish experience. BMC Med Ethics, 17: 49. 27538685 10.1186/s12910-016-0135-z PMC4991003

[80] Klingler C Silva DS Schuermann C (2017). Ethical issues in public health surveillance: a systematic qualitative review. BMC Public Health, 17( 1): 295. 28376752 10.1186/s12889-017-4200-4 PMC5381137

[81] Hassan FI Niaz K Maqbool F (2017). Congenital Abnormalities: Consequence of Maternal Zika Virus Infection: A Narrative review. Infect Disord Drug Targets, 17( 1): 3– 13. 27758685 10.2174/1871526516666161018104916

[82] Bahamondes L Ali M Monteiro I (2017). Contraceptive sales in the setting of the Zika virus epidemic. Hum Reprod, 32( 1): 88– 93. 27932442 10.1093/humrep/dew310 PMC5165082

[83] Shapiro GK (2014). Abortion law in Muslim-majority countries: an overview of the Islamic discourse with policy implications. Health Policy Plan, 29( 4): 483– 94. 23749735 10.1093/heapol/czt040

[84] Levels M Sluiter R Need A (2014). A review of abortion laws in Western-European countries. A cross-national comparison of legal developments between 1960 and 2010. Health Policy, 118( 1): 95– 104. 25059743 10.1016/j.healthpol.2014.06.008

[85] Abbasi M Shamsi Gooshki E Allahbedashti N (2014). Abortion in Iranian legal system. Iran J Allergy Asthma Immunol, 13( 1): 71– 84. 24338232

